# Editorial: Emerging therapies for metabolic bone diseases

**DOI:** 10.3389/fendo.2026.1906569

**Published:** 2026-07-17

**Authors:** Alberto Falchetti, Luigi Gennari

**Affiliations:** 1 Unit of Endocrinology, Santa Maria della Misericordia, Udine, Italy; 2 Universita degli Studi di Siena Dipartimento di Scienze Mediche Chirurgiche e Neuroscienze Sezione di Medicina e Chirurgia, Siena, Italy

**Keywords:** FGF23 signaling, gut - bone axis, metabolic bone diseases, osteoporosis therapies, sclerostin

The field of metabolic bone diseases is undergoing a period of remarkable transformation, driven by advances in molecular biology, the development of targeted therapies, and an increasing appreciation of the complex interactions between the skeleton and multiple extra-skeletal systems. The traditional view of bone as a largely structural tissue has progressively evolved toward a more integrated concept of the skeleton as an active endocrine organ involved in systemic physiological networks, including energy metabolism and immune regulation. In parallel, therapeutic innovation has expanded treatment options for both common disorders such as osteoporosis and rare diseases characterized by profound alterations in mineral metabolism.

The contributions gathered in this Research Topic reflect these developments and provide a comprehensive overview of contemporary advances in bone research, spanning preclinical studies, systematic reviews, real-world evidence, and patient-centered investigations. Collectively, they highlight the multidimensional nature of modern skeletal medicine and the progressive convergence of mechanistic and clinical research.

A central theme emerging from this Research topic is the evolution of pharmacological strategies targeting bone remodeling and mineral metabolism. The antifracture efficacy of zoledronic acid is well established by a large body of clinical evidence accumulated over the past decades, including its use in postmenopausal women with osteopenia, supporting its broad role in fragility fracture prevention. Complementing these clinical data, the preclinical findings in growing animal models, in the paper by Aguirre et al., further refine our understanding of bisphosphonate action, demonstrating that skeletal responses are influenced by age, treatment duration, and skeletal maturity, and underscoring the context-dependent nature of antiresorptive therapy.

Parallel advances have been driven by the elucidation of phosphate metabolism and fibroblast growth factor 23 (FGF23) biology. Insights derived from X-linked hypophosphatemia (XLH), the prototypical FGF23-mediated disorder, have been instrumental in defining the role of phosphate homeostasis in skeletal integrity. Tumor-induced osteomalacia (TIO), an acquired condition caused by ectopic FGF23 production, further exemplifies how dysregulated phosphate handling leads to severe skeletal fragility. As written in the paper by Vergatti et al., the development of burosumab has translated these mechanistic insights into effective targeted therapy, with evidence supporting its use in both XLH and TIO, particularly in cases of delayed tumor localization or surgical inoperability. Nevertheless, emerging data on treatment discontinuation highlight that long-term management strategies remain incompletely defined.

A similar translational pathway is exemplified by sclerostin biology. Its role as a key inhibitor of bone formation was elucidated through the study of rare high bone mass disorders such as sclerosteosis and Van Buchem disease, in which loss of sclerostin function leads to excessive skeletal accrual. These observations established the rationale for anti-sclerostin antibodies, which represent a major advance in osteoporosis therapy through dual stimulation of bone formation and inhibition of resorption associated with both the efficacy and safety, as reported by Chen et al. in their systematic review and meta-analysis of randomized controlled trials. Together with FGF23-related disorders, these examples illustrate how rare skeletal diseases continue to drive therapeutic innovation in more prevalent conditions.

Beyond antiresorptive and anabolic strategies, optimization of mineral metabolism remains clinically relevant. Vitamin D analogues such as doxercalciferol, see the paper by Gallagher et al., continue to be investigated as modulators of bone and calcium homeostasis. While their use in osteoporosis is limited by concerns regarding calcium-related adverse effects, they remain of interest for both their potential therapeutic value and the physiological insights they provide into skeletal regulation, highlighting that refinement of established pathways remains an important avenue of innovation. In this manuscript emerges that doxercalciferol treatment showed no difference from the placebo group on bone loss at 12 months but at 24 months doxercalciferol showed significant prevention of bone loss from the femoral neck.

Beyond pharmacological interventions, Zhong et al. confirm an increasing attention being directed toward systemic determinants of skeletal health. Bone is now recognized as an integrative organ influenced by multiple physiological systems, including the gastrointestinal tract, immune system, and metabolic regulatory networks. In this context, experimental evidence linking gut microbiota dysbiosis to osteoporosis suggests a mechanistic axis involving activation of the local renin–angiotensin system, enhanced osteoclastogenesis, inflammatory signaling, and RANKL-mediated bone resorption. These findings expand the emerging concept of the gut–bone axis and reinforce the systemic nature of skeletal fragility.

Similarly, metabolic and bariatric surgery has emerged as an important determinant of bone health. Sleeve gastrectomy, while highly effective in improving metabolic outcomes in obesity, has been associated with alterations in bone turnover, reductions in bone mineral density, and increased fracture risk. As the global burden of obesity continues to rise, understanding post-surgical skeletal fragility and implementing preventive strategies has become a clinical priority. The review included in this Research Topic, by Fan et al., provides a timely synthesis of current evidence in this rapidly expanding field.

An equally important aspect of contemporary skeletal research concerns patient-reported outcomes and chronic disease burden. In their multicenter survey in chronic hypoparathyroidism, Fuss et al. highlights that, despite conventional calcium and vitamin D therapy, patients continue to experience substantial symptom burden, complications, and impaired quality of life. These findings underscore the limitations of biochemical control as the sole therapeutic endpoint and emphasize the importance of patient-centered outcome measures.

Taken together, the studies included in this Research Topic highlight a clear shift in bone research from descriptive frameworks toward mechanism-driven and pathway-targeted interventions. Whether addressing excess FGF23 activity, modulating sclerostin signaling, or investigating the gut–bone axis, these contributions converge on a common objective: translating advances in bone biology into clinically meaningful strategies.

Despite these important advances, several challenges remain unresolved, including optimal treatment duration, sequencing of therapies, long-term safety, and the management of treatment discontinuation. Furthermore, the increasing availability of highly targeted therapies raises important issues related to accessibility, cost-effectiveness, and equity in healthcare delivery.

In conclusion, this Research Topic illustrates the breadth and dynamism of contemporary bone research. By bridging fundamental mechanisms with clinical application, the contributions provide a more integrated understanding of skeletal biology and a strong foundation for future investigations. We hope that these findings will stimulate further research, foster interdisciplinary collaboration, and ultimately improve outcomes for patients affected by metabolic bone diseases.

